# Synthesis, antioxidant activity, antimicrobial efficacy and molecular docking studies of 4-chloro-2-(1-(4-methoxyphenyl)-4,5-diphenyl-1*H*-imidazol-2-yl)phenol and its transition metal complexes†

**DOI:** 10.1039/d2ra08327b

**Published:** 2023-03-21

**Authors:** Muhammad Saeed Ahmad, Abu Bakar Siddique, Muhammad Khalid, Akbar Ali, Muhammad Ashraf Shaheen, Muhammad Nawaz Tahir, Muhammad Imran, Ahmad Irfan, Muhammad Usman Khan, Marcio Weber Paixão

**Affiliations:** a Institute of Chemistry, University of Sargodha Sargodha 40100 Pakistan ashraf.shaheen@uos.edu.pk; b Institute of Chemistry, Khwaja Fareed University of Engineering & Information Technology Rahim Yar Khan 64200 Pakistan; c Centre for Theoretical and Computational Research, Khwaja Fareed University of Engineering & Information Technology Rahim Yar Khan 64200 Pakistan; d Department of Chemistry, Government College University Faisalabad Faisalabad 38000 Pakistan akbarali@gcuf.edu.pk akbarchm@gmail.com; e Department of Physics, University of Sargodha Sargodha Pakistan; f Department of Chemistry, College of Science, King Khalid University PO. Box 9004 Abha 61413 Saudi Arabia; g Research Center for Advanced Materials Science (RCAMS), King Khalid University PO. Box 9004 Abha 61413 Saudi Arabia; h Department of Chemistry, University of Okara Okara-56300 Pakistan; i Department of Chemistry, Universidade Federal de São Carlos (UFSCar) São Carlos SP Brazil

## Abstract

Herein, a one-pot synthesis of tetra-substituted imidazole, 4-chloro-2-(1-(4-methoxyphenyl)-4,5-diphenyl-1*H*-imidazol-2-yl)phenol (HL), is reported by the reaction of benzil, 5-bromosalicylaldehyde, ammonium acetate and anisidine. The synthesized imidazole was reacted with salts of 1^st^ row transition metals (Co(ii), Ni(ii), Cu(ii), Mn(ii) and Zn(ii)) to obtain metal complexes. The structure of the compounds was confirmed using various spectroscopic and analytical techniques. HL, which is crystalline, was characterized by SC-XRD. Subsequently, the synthesized compounds were evaluated for their antioxidant and antimicrobial activities. Antimicrobial studies revealed the more noxious nature of metal complexes compared to ligand against various strains of bacteria and fungi. Molecular docking results based on the binding energy values also supported the experimental results of the antioxidant activities of the compounds. HL was found to be a better antioxidant than metal complexes. For a better insight into the structure, computational studies of the compounds were also carried out. A clear intra-molecular charge transfer was perceived in the ligand and its metal complexes. The transfer integral values for holes (36.48 meV) were found to be higher than the electron transfer integrals (24.76 meV), which indicated that the ligand would be a better hole transporter. According to the frontier molecular orbitals of the dimer, the charge transfer within the molecule is found from monomer 1 to 2.

## Abstract 2

Herein, a one-pot synthesis of tetra-substituted imidazole, 4-chloro-2-(1-(4-methoxyphenyl)-4,5-diphenyl-1*H*-imidazol-2-yl)phenol (HL), is reported by the reaction of benzil, 5-chlorosalicylaldehyde, ammonium acetate and anisidine.

## Graphical abstract

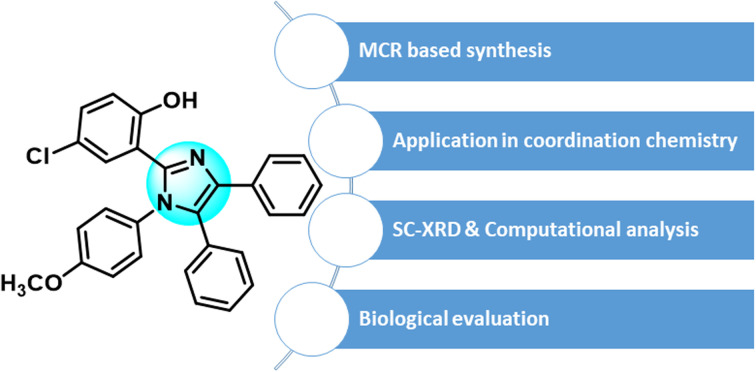

## Introduction

Drug-resistant microbes pose a serious threat to the public's health and are a major cause of concern in the field of medicine.^1^ Among the numerous strategies developed to tackle this issue, the development of new drugs with more activity and different modes of action is a better choice for scientists. Several classes of compounds have been currently evaluated for their antimicrobial and antioxidant activities, such as imines,^2^ alkaloids,^3^ and phenolics.^4^ Among these classes of compounds, imidazole derivatives have gained the increasing attention owing to their diverse applications in the areas of medicinal chemistry and material sciences.^5^

Imidazole is an important class of organic compounds with numerous biological activities, and its derivatives are also found in many chemical and biological systems in nature.^6^ These substituted nitrogenous aromatic compounds are found in many FDA-approved medicines, such as Eprosartan and Losartan.^7^ Imidazole moiety is also found in numerous biological molecules, such as histidine and histamine.^8^ This class of compounds is also known for some other biological activities, such as anti-allergic,^9^ pain relieving,^10^ and anti-inflammatory.^11^ The fluorescent nature of these compounds makes them applicable as whiteners (fluorescence in textiles), optics and photography.^12^ In addition, these scaffolds have found many applications in the field of agriculture, such as pesticides, fungicides, herbicides and plant development regulators.^13^

Owing to the wide applications of this class, various methods have been reported for its synthesis. Tetra-substituted imidazoles are synthesized majorly by reacting vicinal diketone, aldehyde, primary aromatic amine and ammonium acetate. Recently, various catalysts have also been employed, such as iodine,^14^ silica-reinforced propylpiperazine *N*-Sulphamic acid (SBPPSA),^15^ silica-supported NaHSO_4_,^16^ microwave heating,^17^ FeCl_3_·6H_2_O,^18^ heteropoly acids,^19^ Wells–Dawson acid,^20^ DABCO,^21^ ionic liquids,^22^ sodium benzenesulphonate,^23^ strong acid nano-catalyst,^24^ HClO_4_–SiO_2_,^25^ ZrCl_4_,^26^ mercaptopropyl silica (MPS),^27^ BF_3_·SiO_2_ (ref. 28) and biocatalyst, to improve its yields.^29^ However, multicomponent reactions (MCRs) remained a useful strategy owing to great selectivity and high atomic economy.^30^ In MCRs, all the reactants are added simultaneously in a one-pot manner that generally results in reducing the cost and time-effective synthesis of the desired products.^31^

Many organic molecules with nitrogen and oxygen moieties have been employed to treat bacterial and fungal diseases.^32^ Organic compounds and metal frameworks have been known for their potential against resistant strains of bacteria.^33^ These literature investigations indicate that the antimicrobial potential of a drug could be greatly increased after metal complexation.^34^ Metal containing drugs can suppress the growth of both Gram-positive and Gram-negative bacteria.^35^

In this study, substituted imidazole ligand (HL) and its metal complexes (C_1_–C_5_) were synthesized and characterized. The compounds were evaluated against different strains of bacteria and fungi. The antioxidant efficacy of these compounds was evaluated experimentally and compared with the molecular docking results for a better understanding of the interaction of compounds with the NADPH enzyme. The binding energies of synthesized compounds (ligand and its metal complexes) with NADPH (PDB ID-2CDU) enzyme through molecular docking were studied to explore the drug potency, structure–activity relationship and their interaction with the enzyme. Additionally, the inter- and intra-molecular charge transfer nature and transfer integral values of the ligand were also probed by advanced quantum chemical methods.

## Experimental

### Materials and methods

Analytical grade reagents were used in this study without any further purification. The elemental analysis was performed using a PerkinElmer Analyzer. Infrared spectra were recorded using a Shimadzu FTIR-8400S spectrophotometer. A SHIMADZU UV 240 spectrophotometer was used for UV/Visible spectra. A Gallenkamp melting point apparatus was used to measure the melting points. The pre-coated silica gel Aluminium sheets of Merck Company were used for the thin layer chromatography (TLC) to observe the reaction progress. ^1^H-NMR spectrum was recorded in CDCl_3_ by applying Bruker (Rhenistetten-Forchheim, Germany) AM 300 spectrometers, operating at 300 MHz and using TMS as an internal standard. Chemical shifts (*δ*) were reported in parts per million (ppm) and coupling constants in Hz. ^13^C-NMR spectrum was recorded in CDCl_3_ at 75 MHz with the same internal standard. The X-ray diffractometer named Bruker Kappa APEX-II CCD was employed for the intensity data collection of the compound using APEX-II software. The X-ray source of the diffractometer generates Mo Kα radiation with a wavelength of 0.71073 Å. The absorption correction was performed using a multi-scan in SADABS software. The raw intensity data were solved using SHELXS-97 software, whereas for refinement, SHELXL 2018/3 software was employed. For the graphical representation of the SC-XRD results, Mercury 4.0, ORTEP 3, and PLATON programs were used.

### Synthesis of 4-chloro-2-(1-(4-methoxyphenyl)-4,5-diphenyl-1H-imidazol-2-yl)phenol (HL)

**Fig. 1:**
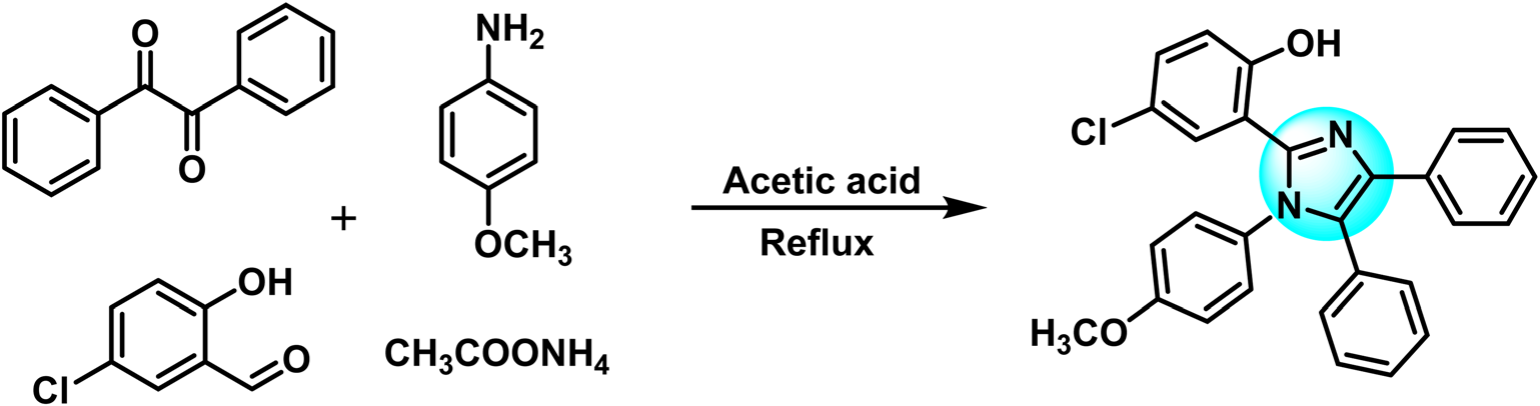
Schematic representation of synthesis of the imidazole ligand.

Benzil (3.0 g, 14.28 mmol) along with 5-chlorosalicylaldehyde (2.24 g, 14.28 mmol) was dissolved in 30 mL of glacial acetic acid at 25 °C. 4-Methoxyaniline (2.63 g, 21.42 mmol) and ammonium acetate (5.7 g, 74 mmol) were also added simultaneously to the mixture. The resulting solution was refluxed for 2.5 hours. The progress of the reaction was continuously checked using TLC. On completion, the dark solution was poured into distilled water. Ethyl acetate was used to extract the substituted imidazole ligand (HL) from the water. The HL was recrystallized in ethyl acetate. Fig. 1 depicts the schematic diagram of the HL.

Yield: 75%; colorless, crystalline; melting point: 153–155 °C; FTIR (KBr, cm^−1^): 3057, 2949 (C–H), 3200–3400 (O–H), 1587 (C=N), 1462 (C=C), 1267 (C–N), 1045 (C–O) (Fig. S3†); *λ*_max_ (cm^−1^): 32 258, 36 231; ^1^H-NMR (300 MHz, CDCl_3_) *δ* (ppm): 3.84 (s, 6H), 6.56 (d, 2H), 6.94 (t, 2H), 7.1–7.35 (m, 8H), 7.57 (d, 5H), 13.55 (s, 1H) (Fig. S1A†); ^13^C-NMR (75 MHz, CDCl_3_) *δ* (ppm): 55.61, 76.67, 77.10, 77.52, 113.95, 114.95, 118.97, 122.60, 125.65, 126.97, 127.24, 128.38, 128.60, 128.63, 129.03, 129.53, 129.71, 131.11, 131.30, 132.71, 134.98, 143.89, 157.08, 160.15. (Fig. S1B†); Elemental analysis: C_28_H_21_ClN_2_O_2_: (452.93), Calculated (%): C; 74.25, H; 4.67, N; 6.18; Found (%): C; 74.32, H; 4.62, N; 6.27.

### Synthesis of transition metal complexes (C_1_–C_5_)

**Fig. 2:**
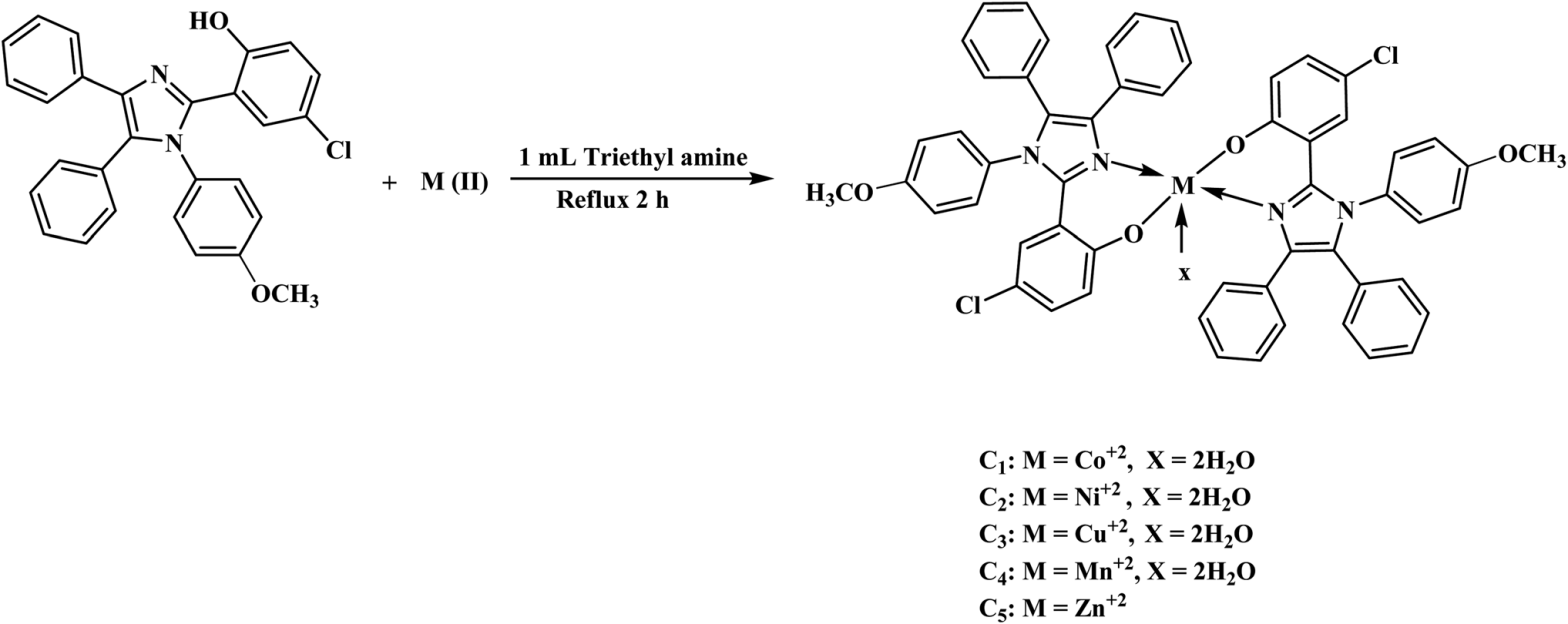
Synthesis of metal complexes of Ligand (HL).

In the ethanolic solution of HL, 0.5 mL of triethylamine was added and stirred for 30 minutes to abstract the proton. Afterward, chlorides of metal, *i.e.*, Co(ii), Ni(ii), Cu(ii), Mn(ii) and Zn(ii) were dissolved in 20 mL of C_2_H_5_OH and added to HL solution with constant stirring for 2 h. The ligand-to-metal ratio was adjusted to 2 : 1. The formation of colored precipitates adhered to the walls of the flask indicated the formation of a metal complex. The colored precipitates were filtered with sintered glass and dried in a desiccator for 24 h. The dried precipitates were stored in bottles. Fig. 2 and Scheme S1† demonstrate the synthesis of transition metal complexes (C_1_–C_5_).

### Cobalt(ii) complex (C_1_)

Yield: 79%; light brown amorphous solid; melting Point/decomposition temperature: 335–337 °C; FTIR (KBr, cm^−1^): 3056 (C–H), 2343, 1583 (C=N), 1483 (C=C), 1282 (C–N), 715 (M ← O), 453 (M ← N) (Fig. S4†); *λ*_max_ (cm^−1^): 15 427, 17 760, 20 272; Conductance (μS cm^−1^) = 0.96; μeff (B.M.) = 1.7; Elemental Analysis: C_56_H_40_Cl_2_N_4_O_4_Co (964.8) Calculated (%): C; 69.65, H; 4.14, N; 5.80; Found (%): C; 69.87, H; 4.18, N; 5.91.

### Nickel(ii) complex (C_2_)

Yield: 76%; grey amorphous solid; melting point/decomposition temperature: 326–328 °C; FTIR (KBr, cm^−1^): 3050 (C–H stretch), 1592, (C=N), 1487 (C=C), 1360 (C–O), 1274 (C–N stretch), 734 (M ← O), 470 (M ← N) (Fig. S5†); *λ*_max_ (cm^−1^): 12 790, 18 285, 27 403; Conductance (μS cm^−1^) = 0.77; μeff (B.M.) = 3.2; Elemental Analysis: C_56_H_40_Cl_2_N_4_O_4_Ni (964.5) Calculated (%): C; 69.67, H; 4.14, N; 5.80; Found (%): C; 69.54, H; 4.38, N; 5.47.

### Copper(ii) complex (C_3_)

Yield: 86%; light green amorphous solid; melting point: 316–318 °C (decomp.); FTIR (KBr, cm^−1^): 3049 (C–H stretch), 1582, (C=N), 1493 (C=C), 1276 (C–N stretch), 740 (M ← O), 532 (M ← N) (Fig. S6†); *λ*_max_ (cm^−1^): 15 278–15360; Conductance (μS cm^−1^) = 1.07; μeff (B.M.) = 1.9; Elemental Analysis: C_56_H_40_Cl_2_N_4_O_4_Cu (969.3) Calculated (%): C; 69.30, H; 4.12, N; 5.77; Found (%): C; 69.49, H; 4.26, N; 5.37.

### Manganese(ii) complex (C_4_)

Yield: 79%; dusty pink amorphous solid; melting Point: 327–329 °C (decomp.); FT-IR (KBr, cm^−1^): 3054 (C–H stretch), 1584 (C=N), 1490 (C=C), 1378(C–O), 1265 (C–N stretch), 731 (M ← O), 484 (M ← N); *λ*_max_ (cm^−1^): 16 623, 20 748, 24 421, 27 203; Conductance (μS cm^−1^) = 0.94; μeff (B.M.) = 1.8; Elemental Analysis: C_56_H_40_Cl_2_ N_4_O_4_Mn (960.8) Calculated (%): C; 69.94, H; 4.16, N; 5.82; Found (%): C; 69.73, H; 4.32, N; 5.47.

### Zinc(ii) complex (C_5_)

Yield: 82%; white amorphous solid; melting point: 314–316 °C (decomp.); FTIR (KBr, cm^−1^): 3053 (C–H stretch) 1596 (C=N), 1487 (C=C), 1269 (C–N stretch), 711 (M ← O), 538 (M ← N) (Fig. S8†); *λ*_max_ (cm^−1^): 28 340; Conductance (μS cm^−1^) = 1.05; μeff (B.M.) = 0, diamagnetic; Elemental Analysis: C_56_H_40_Cl_2_ N_4_O_4_Zn (971.2) Calculated (%): C; 69.19, H; 4.11, N; 5.76; Found (%): C; 69.76, H; 4.69, N; 5.28.

## SC-XRD analysis

**Fig. 3:**
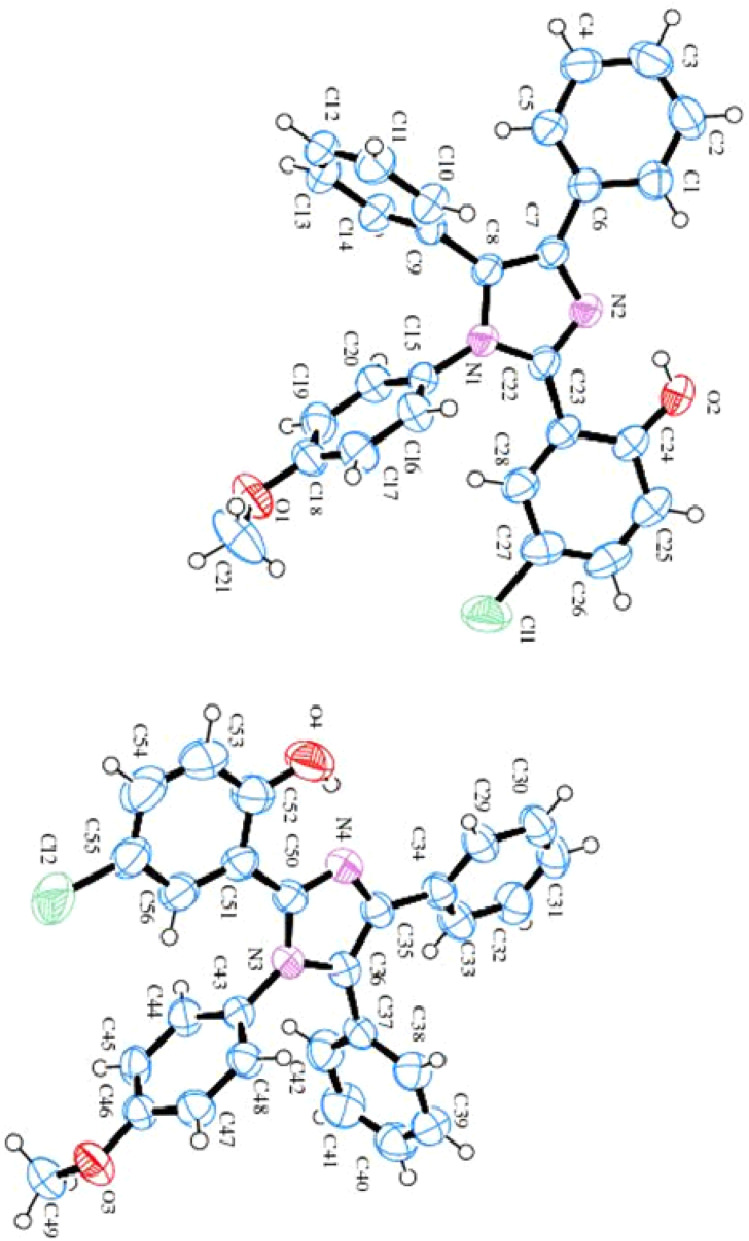
ORTEP diagram of ligand HL.

Single crystal X-ray diffraction data for 4-chloro-2-(1-(4-methoxyphenyl)-4,5-diphenyl-1*H*-imidazol-2-yl)phenol (HL) comprises two independent molecules (as shown in Table 1 and Fig. 3). In one molecule, two phenyl substituents A (C1–C6) and B (C9–C14), the imidazole moiety C (C7/C8/N1/C22/N2), the methoxy-benzene ring D (C15–C21/O1) and 4-chloro-phenol substituent E (C23–C28/O2/Cl1) are planar with r. m. s. along with deviance of 0.0078, 0.0015, 0.0003, 0.0042 and 0.0058 Å. The dihedral angle amongst A/B, A/C, A/D, A/E, B/C, B/D, B/E, C/D, C/E and D/E are 75.347(0.065)°, 28.391(0.091)°, 58.466(0.052)°, 22.773(0.095)°, 76.354(0.063)°, 69.652(0.067)°, 71.871(0.064)°, 82.885(0.053)°, 7.158(0.102)° and 79.636(0.051)°, respectively. In the molecule, O–H⋯N hydrogen bonding interactions constitute the S (6) ring. In the second molecule, the phenyl substituent F (C29–C34), G (C37–C42), the imidazole circle H (C35/C36/N3/C50/N4), the methoxy-benzene ring I (C43–C49/O3) and 4-chloro-phenol ring J (C51–C56/O4/Cl2) are planar with r. m. s. having deviations of 0.0039, 0.0067, 0.0031, 0.0033 and 0.0043 Å, respectively. The dihedral angle appearing among F/G, F/H, F/I, F/J, G/H, G/I, G/J, H/I, H/J and I/J is 66.555 (0.072)°, 27.665 (0.046)°, 67.431 (0.070)°, 27.287 (0.050)°, 89.336 (0.061)°, 71.280 (0.070)°, 73.751 (0.066)°, 68.336 (0.077)°, 2.954 (0.086)° and 88.242 (0.064)°, respectively. This molecule also has S (6) rings formed because of O–H⋯N HB interactions. Fig. S2† illustrates the molecular packing of ligand HL.

**Table 1:** Crystallographic data of novel ligand (HL)

Crystal data	Imidazole ligand (HL)
CCDC	2041734
Chemical formula	C_28_H_21_ClN_2_O_2_
*M*_r_	452.92
Crystal system, space group	Triclinic, *P*1̄
Temperature (K)	296
*a*, *b*, *c* (Å)	9.5810 (9), 12.8164 (13), 19.359 (2)
*α*, *β*, *γ* (°)	89.075 (4), 84.551 (3), 77.443 (4)
*V* (Å^3^)	2309.8 (4)
*Z*	4
Radiation type	Mo *K*α
*μ* (mm^−1^)	0.19
No. of measured, independent and observed [*I* > 2*σ*(*I*)] reflections	27 856, 11 202, 6353
*R*_int_	0.046
(Sin *θ*/*λ*)_max_ (Å^−1^)	0.665
Refinement	
*R*[*F*^2^ > 2*σ*(*F*^2^)], *wR*(*F*^2^), *S*	0.050, 0.147, 1.01
No. of reflections	11 202
No. of parameters	599
H-atom treatment	H-atom parameters constrained
*Δ*_max_, *Δ*_min_ (e Å^−3^)	0.37, −0.35

### Antioxidant screening of synthesized ligand and metal complexes

The antioxidant properties of the synthesized compounds were determined by DPPH and ABTS assays.

### DPPH antioxidant activity

The antioxidant potential of compounds was evaluated by DPPH assay following the standard method described by Blois^36^ and Desmarchelier *et al.*^37^ Standard solutions of various concentrations of standards (Trolox and BHA) and synthesized compounds were prepared in DMSO to determine the MIC value. 4 mL of 0.1 nM DPPH solution in ethanol was added to the 1 mL standard solutions (10–100 μM) of each synthesized compound. These solutions were kept for 30 minutes in the dark at 25 °C. After baseline correction using ethanol, the decrease in absorbance (*A*_0_ − *A*_1_) was determined for each solution at *λ*_max_ of 517 nm. The following equation was used to measure the activity:Antioxidant activity (%) = [(*A*_0_ − *A*_1_)/*A*_0_] × 100.

### ABTS antioxidant activity

The antioxidant potential of the compounds was also evaluated by the ABTS scavenging capacity of the synthesized compounds.^38^ The ABTS radical was liberated by the reaction of potassium persulphate and ABTS in a 1 : 1.25 ratio. The mixture was incubated at 25 °C for 12 h in the dark. 1 mL of ABTS radical solution was added to 3 mL solution of each synthesized compound in the concentration range of 10–100 μM. The decrease in absorbance (*A*_c_ − *A*_s_) for each solution was measured at 734 nm after an incubation period of 30 minutes in the dark. The following equation was used to determine the percentage of ABTS radical scavenging capacity:ABTS scavenging capacity (%) = [(*A*_c_ − *A*_s_)/*A*_c_] × 100.

### Antimicrobial screening of ligand (HL) and metal complexes

The antimicrobial efficacy of compounds was evaluated against three bacterial strains (*Escherichia coli*, *Klebsiella pneumoni* and *Staphylococcus aureus*) and three fungal strains (*Candida glabrata*, *Candida albicans* and *Candida krusei*) using the broth dilution method.^39^ The broad-spectrum drugs Chloramphenicol and Ketoconazole were used as positive controls for antibacterial and antifungal activity, respectively. MIC value of each compound was calculated for each bacterial and fungal strains using the broth microdilution method.

#### Broth micro dilution assay

Cultures of bacterial and fungal strains were prepared in Mueller–Hinton and Sabouraud dextrose broth by incubation at 35 °C for 24 h, respectively. Dilute solutions of each synthesized compound and standard drug in the concentration range of 800–1.56 μg mL^−1^ were prepared in DMSO. MIC value (μg mL^−1^) of each sample was calculated thrice at pH 7 in Mueller–Hinton and Sabouraud dextrose broth.

### Molecular docking studies

In this study, Autodock version 4.2 with Autodock tools and MGL tools was used to investigate the binding energy values. The H_2_O molecules were removed, and polar hydrogen atoms were added to the structures of the compounds. The docking output files were opened in Pymol version 1.7.4.5 Edu to investigate the interaction between the drug and enzyme. It is important to note that no docking study of these drugs with the NADPH (PDB ID-2CDU) enzyme was carried out earlier. Thus, we explored their interaction behavior through *in silico* analysis. Correlations of the synthesized compound crystal structure of NADPH were investigated. Molecular docking was documented by the insertion of synthesized compounds (ligands and metal complexes (C_1_–C_5_)) within the crystal structure binding site of NADPH to gain a better understanding of drug potency and structure–activity relationship.

### Statistical analysis

Triplicate analysis was carried out for each bioactivity test in this study. The results were analyzed statistically using ANOVA. Statistical significance was accepted at a level of *p* < 0.05. All the results are reported as mean ± SD.

## Results and discussions

HL was synthesized by the multicomponent reaction of benzil, 5-chlorosalicylaldehyde, 4-methoxyaniline and ammonium acetate. HL was characterized by FT-IR, UV-visible spectroscopy, ^1^H-NMR, ^13^C-NMR, SC-XRD and elemental analysis. In the FT-IR spectra, the formation of ligand was indicated by the appearance of peaks at 3200–3400, 1587, 1462 and 1262 cm^−1^ attributed to the stretching vibrations of –OH, C=N, aromatic C=C and C–N bonds, respectively. Electronic transitions at 32 258 and 36 231 cm^−1^ were assigned to the π → π* and n → π* transitions in HL, respectively. Shifts in ^1^H-NMR and ^13^C-NMR spectra and SC-XRD parameters were examined to confirm the synthesis and structure elucidation of HL. This compound also exhibits fluorescence under UV light owing to ESIPT (excited state intra-molecular proton transfer) from the hydroxyl group to the imidazole ring.

Transition metal complexes of HL (C_1_–C_5_) with 1^st^ row transition metals were synthesized by the reaction of HL and metal salts with a mole ratio of 2 : 1 in the presence of triethylamine as the base for the abstraction of a proton from the –OH group. The disappearance of the broad peak of the –OH group in 3200–3400 cm^−1^ and the formation of new peaks in the region of 400–750 cm^−1^ because of M−N and M−O bonds confirmed the synthesis of metal complexes. Moreover, the appearance of absorption bands in the visible region due to d–d transitions and the charge transfer phenomenon also confirmed the coordination of HL with metals.

Octahedral geometries of manganese, nickel, cobalt and copper were suggested based on electronic spectra supported using the effective magnetic moment data. The low-spin octahedral geometry of the Co(ii) complex was confirmed by an effective magnetic moment value of 1.7 B.M. and electronic absorption bands around 15 427, 17 760 and 20 272 cm^−1^ due to the transitions ^4^T_1g_(F) → ^4^T_2g_(F), ^4^T_1g_(F) → ^4^T_1g_(P) and ^4^T_1g_ (F) → ^4^A_2g_ (F), respectively. In the electronic spectra of the Ni(ii) complex, the presence of three absorpti on peaks at 12 790, 18 285 and 27 403 cm^−1^ due to ^3^A_2g_(F) → ^3^T_2g_(F), ^3^A_2g_(F) → ^3^T_1g_(F) and ^3^A_2g_(F) → ^3^T_2g_(P), respectively, transitions and an effective magnetic moment value of 3.2 B.M. confirmed the octahedral symmetry of the complex. The Cu(ii) complex exhibited an effective magnetic moment value of 1.9 B.M. and a single absorption broad band range of 15 278–15 360 cm^−1^ owing to ^2^E_g_ → ^2^T_2g_ transition. These parameters hinted at the octahedral symmetry of the Cu(ii) complex. Similarly, the octahedral geometry of the Mn(ii) complex was predicted based on the effective magnetic moment value (1.8 B.M.) and four absorption peaks at 16 623, 20 748, 24 421 and 27203 cm^−1^ due to ^4^T_1g_(G) → ^4^T_2g_(G), ^4^T_2g_(G) → ^4^E_g_(G), ^4^T_1g_(G) → ^4^A_1g_, and ^4^E_g_(D) → ^4^T1_g_(P), respectively, transitions in the absorption spectra of the complex. The absorption spectrum of the Zn(ii) complex showed the absorption of light in the UV region at 28 340 cm^−1^. The effect of the magnetic moment proved the diamagnetic nature of the complex.

### Antioxidant activity

HL and its complexes showed moderate antioxidant activities (as shown in Table 2). However, the antioxidant activity of ligand (HL) was found to be better than that of complexes because of the presence of exchangeable hydrogen in the hydroxyl group. Generally, compounds containing hydroxyl groups have the better radical scavenging ability, *i.e.*, phenolics and flavonoids. The percent activities of the compounds were measured using TROLOX and BHA as positive controls. It was also found that the synthesized compounds showed more activity against the DPPH assay compared to the ABTS assay.

**Table 2:** Anti-oxidant activity of compounds

Compound	Radical scavenging activity (%)	
	DPPH assay	ABTS assay
HL	33 ± 1	28 ± 0.4
C_1_	24 ± 2	21 ± 0.5
C_2_	17 ± 0.5	15 ± 1.5
C_3_	23 ± 2	18 ± 1
C_4_	18 ± 0.2	14 ± 0.5
C_5_	27 ± 3	23 ± 2
BHA	78.5 ± 0.5	75.6 ± 0.5
TROLOX	85.5 ± 0.8	88.3 ± 0.7

### Antimicrobial activity

The antimicrobial activity results of HL and its metal complexes are presented in terms of MIC values (μg mL^−1^), as illustrated in Table 3. These results show that the tested compounds have variable inhibitory effects against antibacterial and antifungal strains. Metal complexes showed better antimicrobial activity than HL. The antimicrobial activities of compounds may be attributed to the presence of imidazole rings and chloro-substituents in moieties. A better antimicrobial activity of complexes was attributed to the fact that metal ions can also inhibit the growth of microorganisms by interacting with proteins to hinder their normal functioning if they cross the membrane barriers. Metal ions can easily cross the membrane barriers on complexation because of the enhanced lipophilicity of complexes than salts. It was also observed that Zinc and Copper complexes were more active than others.

**Table 3:** MIC values (μg mL^−1^) of compounds against bacterial and fungal strains^a^

Compounds	*A*	*B*	*C*	*D*	*E*	*F*
HL	25 ± 0.7	50 ± 0.6	50 ± 0.6	50 ± 0.8	100 ± 0.9	100 ± 0.9
C_1_	12.5 ± 0.2	6.25 ± 0.1	6.25 ± 0.2	25 ± 0.5	25 ± 0.5	50 ± 0.4
C_2_	12.5 ± 0.1	12.5 ± 0.3	6.25 ± 0.4	25 ± 0.3	25 ± 0.2	50 ± 0.6
C_3_	6.25 ± 0.4	6.25 ± 0.4	6.25 ± 0.2	12.5 ± 0.4	12.5 ± 0.3	12.5 ± 0.4
C_4_	25 ± 0.3	12.5 ± 0.2	12.5 ± 0.4	25 ± 0.6	50 ± 0.5	50 ± 0.3
C_5_	6.25 ± 0.1	3.125 ± 0.1	3.125 ± 0.1	12.5 ± 0.3	12.5 ± 0.1	6.25 ± 0.2
Ref-1	3.125 ± 0.1	3.125 ± 0.1	3.125 ± 0.1	—	—	—
Ref-2	—	—	—	6.25 ± 0.2	6.25 ± 0.1	3.125 ± 0.1

a A: *Staphylococcus aureus*, B: *Klebsiella pneumonia*, C: *Escherichia coli* D: *Candida glabrata*, E: *Candida albicans*, F: *Candida krusei*, Ref-1: Chloramphenicol, Ref-2: Ketoconazole.

### Molecular docking studies of compound interactions with NADPH

**Fig. 4:**
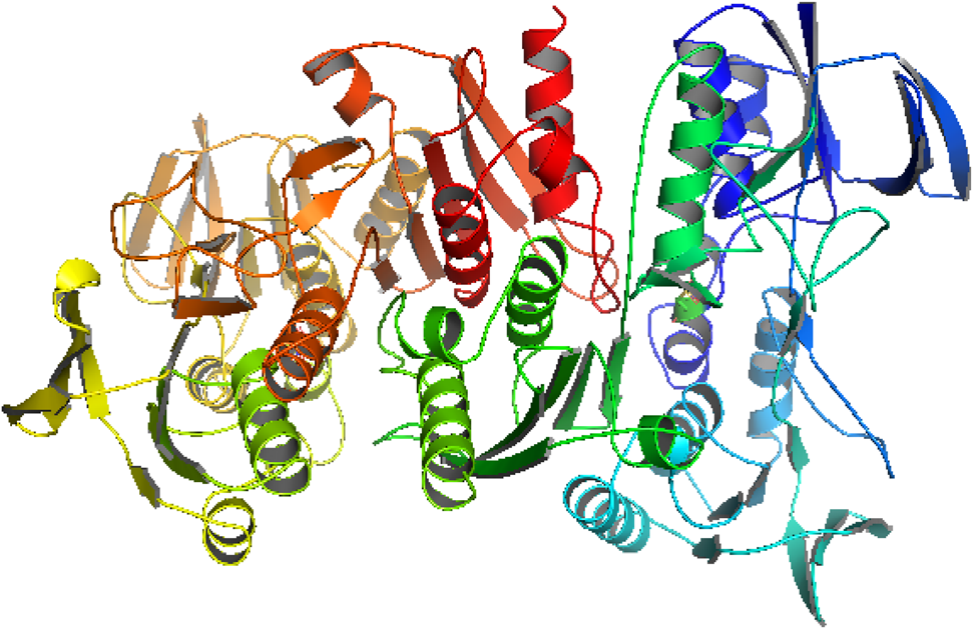
Crystal structure of the NADPH (PDB ID-2CDU) enzyme.

A molecular docking study is an important parameter for better understanding the interaction of bioactive agents with biomolecules.^40^ NADPH (PDB ID-2CDU) is an essential co-enzyme of oxidoreductases and is majorly involved in the production of reactive oxygen species (ROS). ROS cause oxidative stress damage associated with many chronic diseases, such as cancer, diabetes, and asthma. Hence, the inhibition of NADPH-dependent enzymes is highly desirable to control oxidative stress. Therefore, we targeted this protein for *in silico* studies to better understand the insight of the antioxidant activity of synthesized compounds by interaction with NADPH protein.^41^ The structure of the 2CDU NADPH enzyme was obtained from the worldwide Protein Data Bank. Fig. 4 illustrates the 2CDU NADPH enzyme crystal structure without H_2_O and inhibitor molecules. The active sites between the enzyme and drugs are illustrated in Fig. S9.† The molecular docking results showed binding energy values of −6.37, −5.16, −3.58, −5.02, −3.31 and −5.59 for ligand (HL) and metal complexes (C_1_–C_5_) with NADPH enzyme. These values suggest that the ligand (HL) has better antioxidant potential than complexes (C_1_–C_5_). These findings also support the experimental result data of antioxidant activities, *i.e.*, ABTS radical scavenging activity of metal complexes C_1_–C_5_ is smaller than the ligands.

### Charge transfer

Based on the particle diffusion technique, the Brownian motion process can be written as charge transport (CT)^42^ and amalgamated with Marcus theory to conduct the self-exchange reaction process (SERP) for several electron-transfer (ET), as shown in the following equation:^43^

1 *K* = *V*^2^/*h*(π/*λk*_B_*T*)^1/2^ exp (−*λ*/3*k*_B_*T*)

Two major parameters control the ET rate within the SERP, *i.e.*, transfer integral (*V*) and reorganization energy (*λ*); *h* is Planck's constant. The transfer integral *V* values for hole and electron were estimated by the ADF package using a good quality fragment approach. The methodology used to calculate the different parameters is briefly explained in the calculation of charge transport (CT) and transfer integral (*V*) in the ESI.†

The transfer integral for the hole (36.48 meV) was superior to the electron transfer integral value (24.76 meV) when the monomer-to-monomer distance was 12.066 Å. In the dimer, intermolecular charge transfer was perceived from monomer 1 to 2, as shown in Fig. S10.† It was found that the metal to charge phenomenon occurs in compounds C_1_ and C_2_ while ligand to ligand and ligand to metal charge transfer phenomena occur in compound C_3_ and compounds (C_4_ and C_5_), respectively, as shown in Fig. S11.†

## Conclusion

A novel imidazole derivative (HL) and its metal complexes (C_1_–C_5_) were synthesized and characterized by different spectroscopic and analytical techniques. These compounds were evaluated for their antioxidant and antimicrobial potential. The antioxidant activity of the ligand was found to be better than that of its complexes because of the presence of hydroxyl groups in the ligand (HL). Antimicrobial study data showed a more noxious nature of metal complexes compared to the ligand (HL). Metal complexes of zinc and copper were more active antimicrobial agents against different strains of bacteria and fungi compared to the other metal complexes. The transfer integral of the hole (36.48 meV) was found to be higher than that of the electron (24.76 meV), indicating that the ligand may be the better hole transport carrier. According to the frontier orbitals of the dimer, intermolecular charge transfer within a molecule is found from monomer 1 to 2. Molecular docking studies of all synthesized compounds also supported the experimental results of the antioxidant activity. Binding strength values showed that the ligand may be a better antioxidant competitor compared to its metal complexes, which agrees well with the experimental values.

## X-ray Crystallography

Deposition Numbers 2041734 for imidazole ligand contain the supplementary crystallographic data for this paper. These data are provided free of charge by the joint Cambridge Crystallographic Data Centre and Fachinformationszentrum Karlsruhe Access Structures service.

## Author contributions

Muhammad Saeed Ahmad: conceptualization, data curation, writing – original draft preparation. Abu Bakar Siddique: antimicrobial studies, data curation, writing – original draft preparation. Muhammad Khalid: data curation, methodology, software. Akbar Ali: conceptualization, data curation, writing – original draft preparation. Muhammad Ashraf Shaheen: conceptualization, writing – reviewing and editing. Muhammad Nawaz Tahir: methodology, software conceptualization. Muhammad Imran: conceptualization, writing – reviewing and editing, resources. Ahmad Irfan: methodology, supervision, conceptualization. Muhammad Usman Khan: methodology, visualization, writing – original draft preparation. Marcio Weber Paixão: visualization, writing – original draft preparation.

## Conflicts of interest

The authors declare that they have no known competing financial interests or personal relationships that could have appeared to influence the work reported in this paper.

## Supplementary Material

RA-013-D2RA08327B-s001

RA-013-D2RA08327B-s002
