# Supplementary material for: Synthesis, antioxidant activity, antimicrobial efficacy and molecular docking studies of 4-chloro-2-(1-(4-methoxyphenyl)-4,5-diphenyl-1H-imidazol-2-yl)phenol and its transition metal complexes
Source: RSC Adv. 2023 Mar 21;13(14):9222–30. doi: 10.1039/d2ra08327b (PMC10029809; doi:10.1039/d2ra08327b)
Supplement: RA-013-D2RA08327B-s001 [file RA-013-D2RA08327B-s001.pdf]

Electronic Supplementary Material (ESI) for RSC Advances.
This journal is © The Royal Society of Chemistry 2023

Supplementary material

Synthesis, Antioxidant Activity, Antimicrobial Efficacy and Molecular Docking Studies of 4-chloro-2-(1-(4-methoxyphenyl)-4,5-diphenyl-1H-imidazol-2-yl)phenol and its Transition Metal Complexes

Muhammad Saeed Ahmad,[a] Abu Bakar Siddique,[a] Muhammad Khalid,[b,c] Akbar Ali,*[d] Muhammad Ashraf Shaheen,*[a] Muhammad Nawaz Tahir,[e] Muhammad Imran,[f] Ahmad Irfan,[f,g] Muhammad Usman Khan,[b,h] Marcio Weber Paixão[i]

[a] *Department of Chemistry, University of Sargodha, Sargodha, Pakistan*

[b] *Institute of Chemistry, Khwaja Fareed University of Engineering & Information Technology, Rahim Yar Khan, 64200, Pakistan*

[c] *Centre for Theoretical and Computational Research, Khwaja Fareed University of Engineering & Information Technology, Rahim Yar Khan, 64200, Pakistan*

[d] *Department of Chemistry, Government College University Faisalabad, Faisalabad-38000, Pakistan.*

[e] *Department of Physics, University of Sargodha, Sargodha, Pakistan*

[f] *Department of Chemistry, College of Science, King Khalid University, PO. Box 9004, Abha 61413, Saudi Arabia*

[g] *Research Center for Advanced Materials Science (RCAMS), King Khalid University, PO. Box 9004, Abha 61413, Saudi Arabia*

[h] *Department of Chemistry, University of Okara, Okara-56300, Pakistan*

[i] *Department of Chemistry, Universidade Federal de São Carlos (UFSCar), São Carlos, SP, Brazil.*

*Corresponding authors E-mail addresses:

Akbar Ali: akbarchm@gmail.com & akbarali@gcuf.edu.pk

Muhammad Ashraf Shaheen Email: ashraf.shaheen@uos.edu.pk

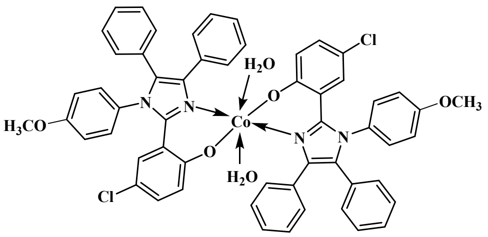

Cobalt(II) Complex **(C_1)**

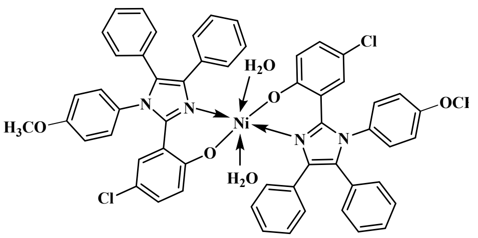

Nickel(II) Complex **(C_2)**

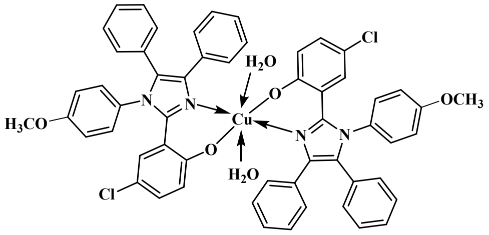

Copper(II) Complex **(C_3)**

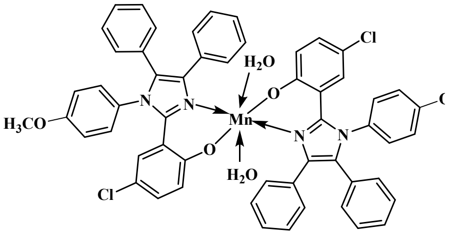

Manganese(II) Complex **(C_4)**

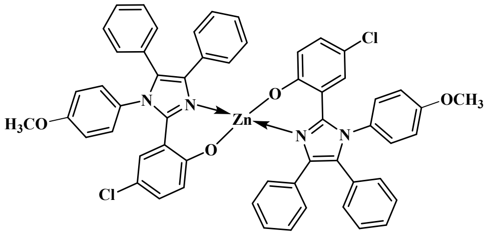

Zinc(II) Complex **(C_5)**

Scheme S1: Structures of Transition Metal Complexes **(C_1-C_5)**

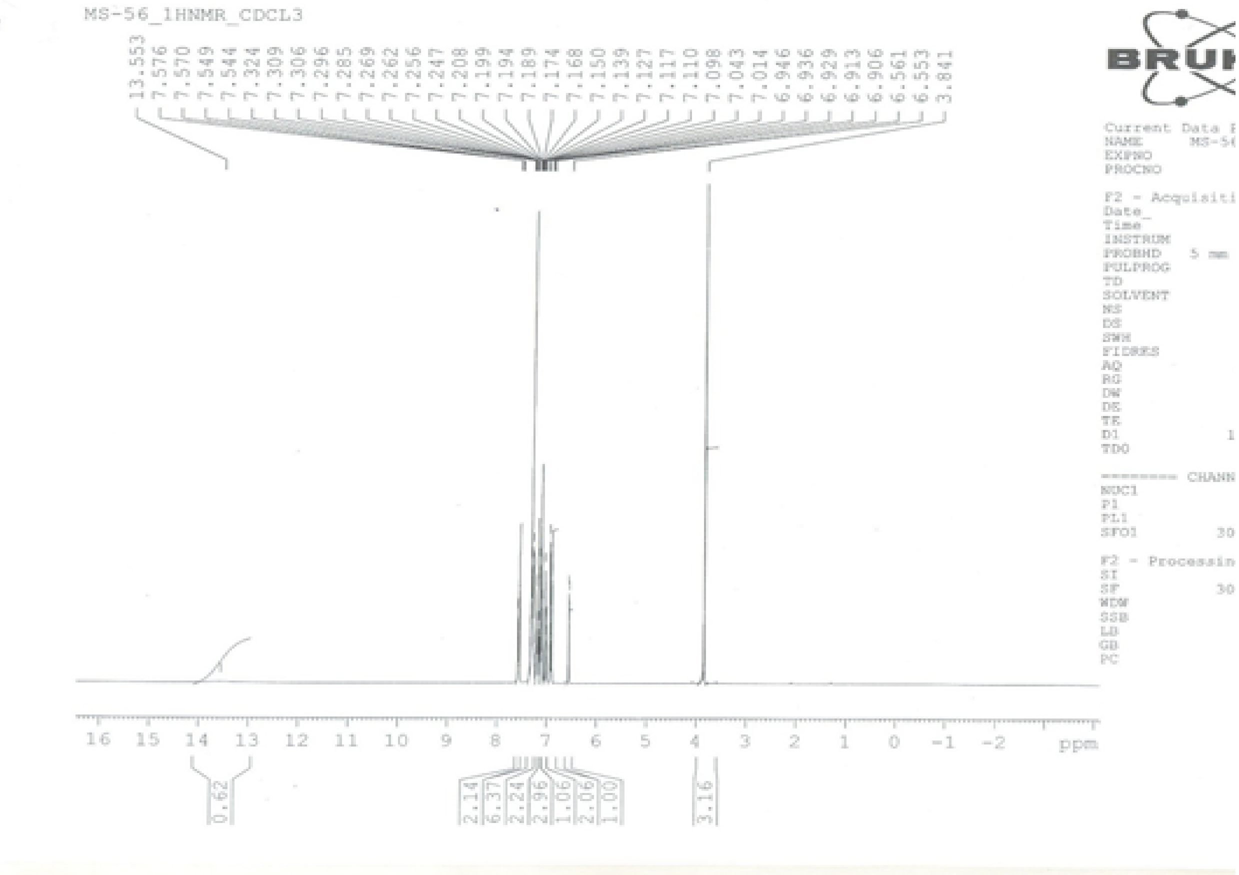

Fig. S1 A: ^{1}H-NMR spectra of *4-chloro-2-(1-(4-methoxyphenyl)-4,5-diphenyl-1H-imidazol-2-yl)phenol* (**HL**)

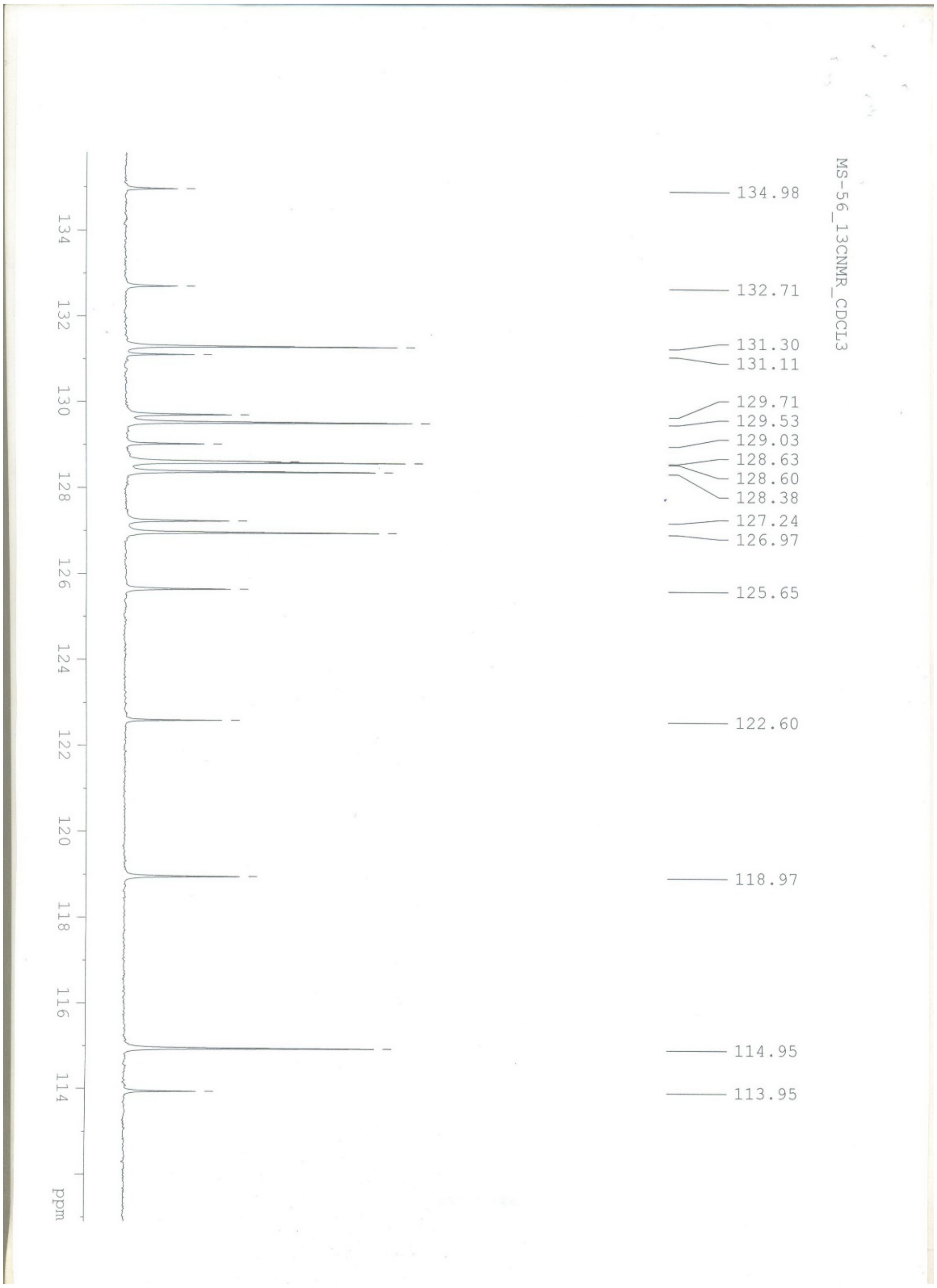

Fig. S1 B: ^{13}C-NMR spectra of *4-chloro-2-(1-(4-methoxyphenyl)-4,5-diphenyl-1H-imidazol-2-yl)phenol* (HL)

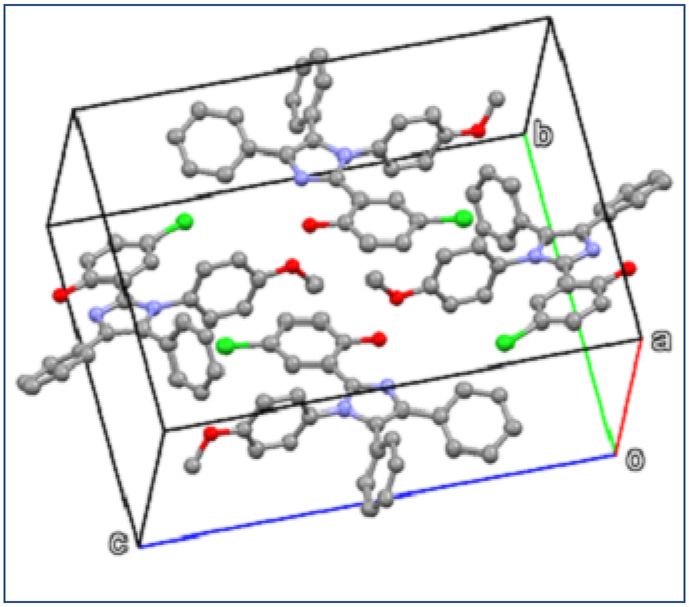

Fig. S2: Molecular packing of ligand (**HL**)

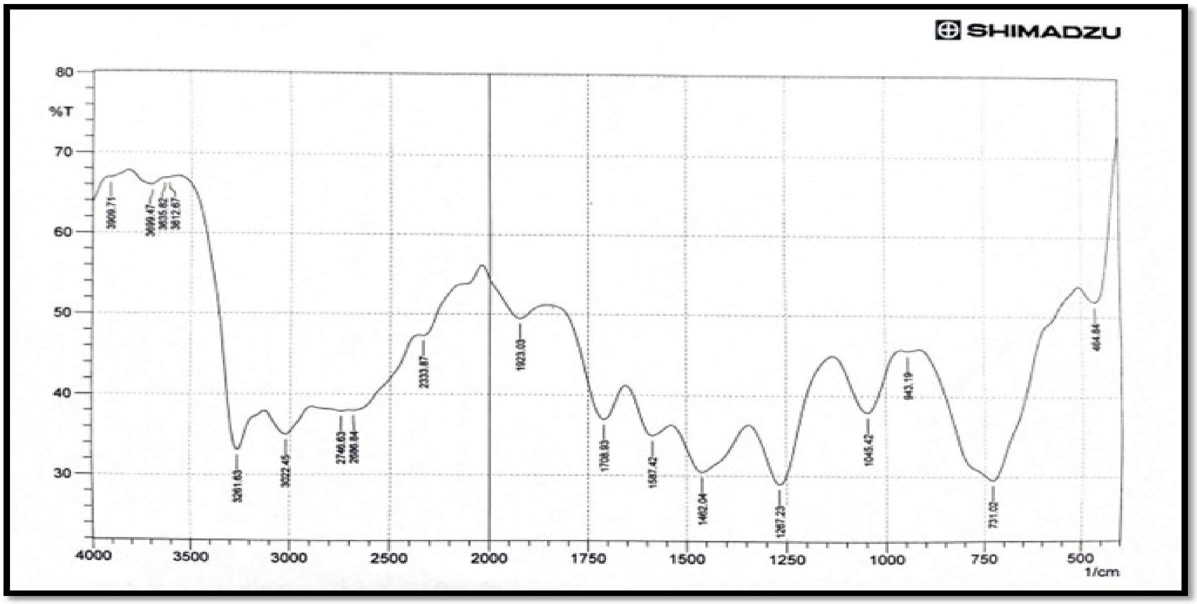

Fig. S3: FTIR Spectra of Ligand (**HL)**

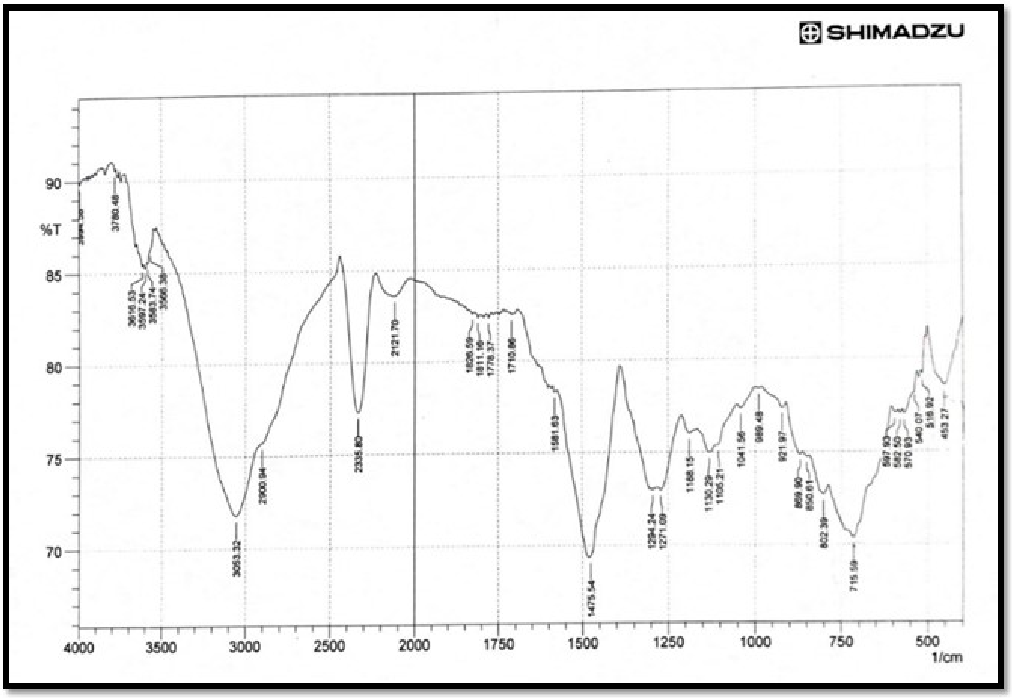

Fig. S4: FTIR Spectrum of *Cobalt (II) complex (*C_1*)*

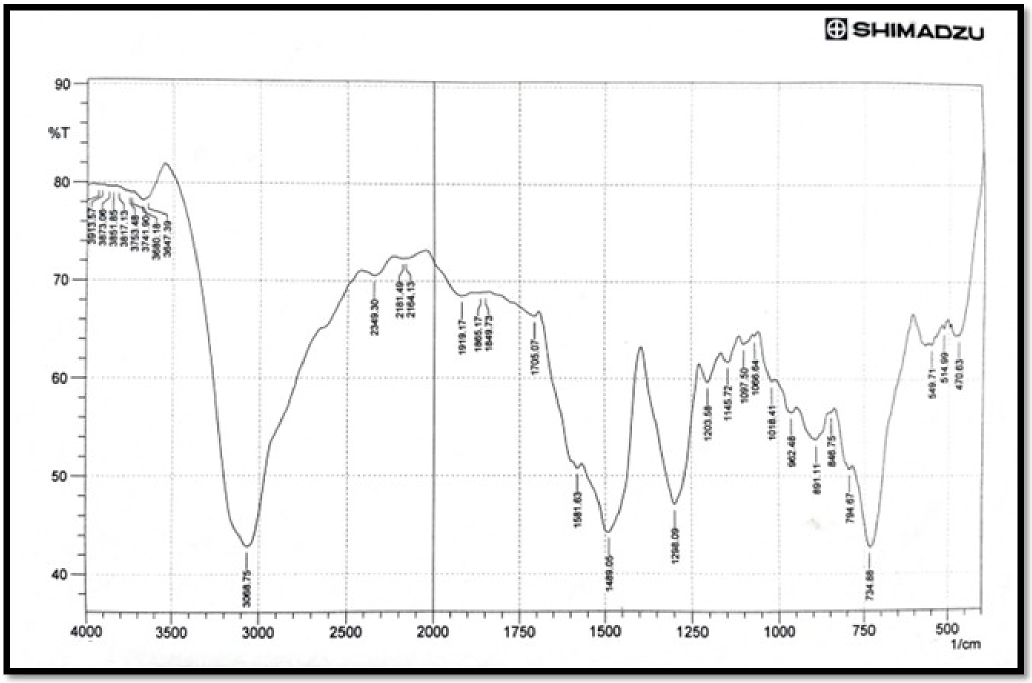

Fig. S5: FTIR Spectrum of *Nickel (II) complex (C_2)*

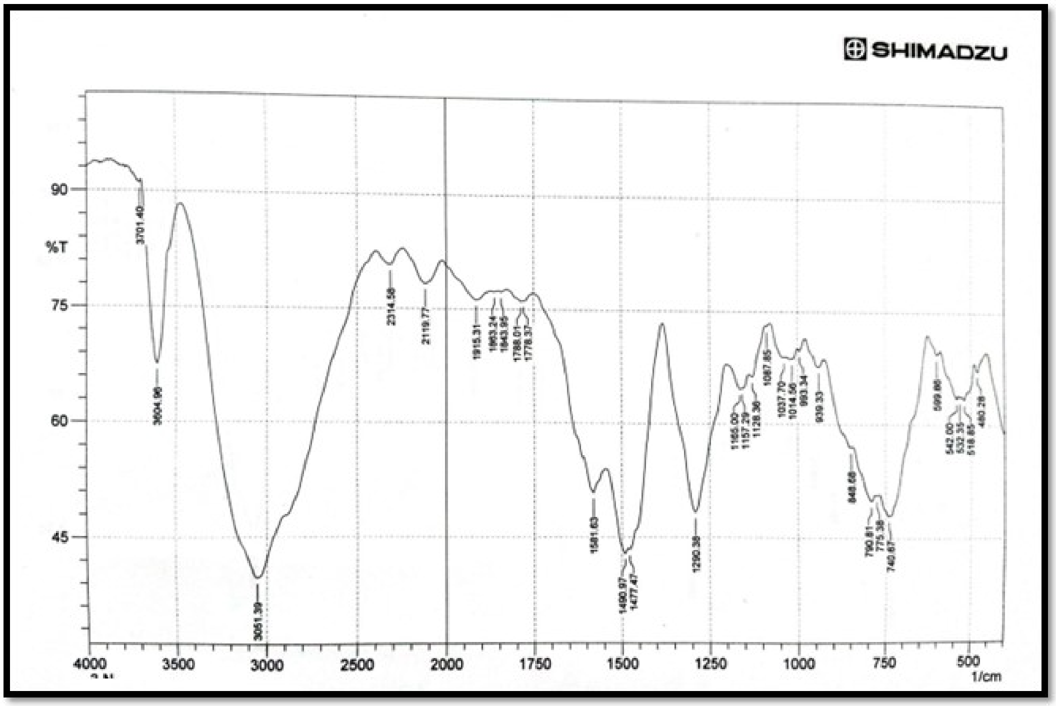

Fig. S6: FTIR Spectrum of *Copper (II) complex (C$_3$)*

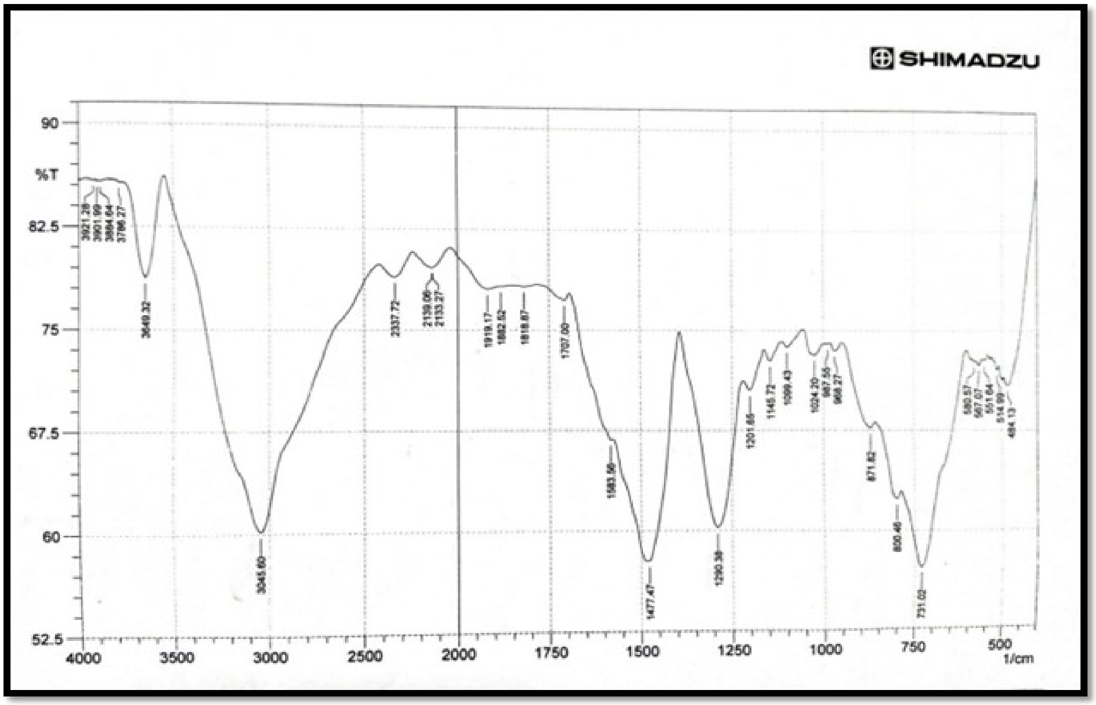

Fig. S7: FTIR Spectrum of *Manganese (II) complex (C_4)*

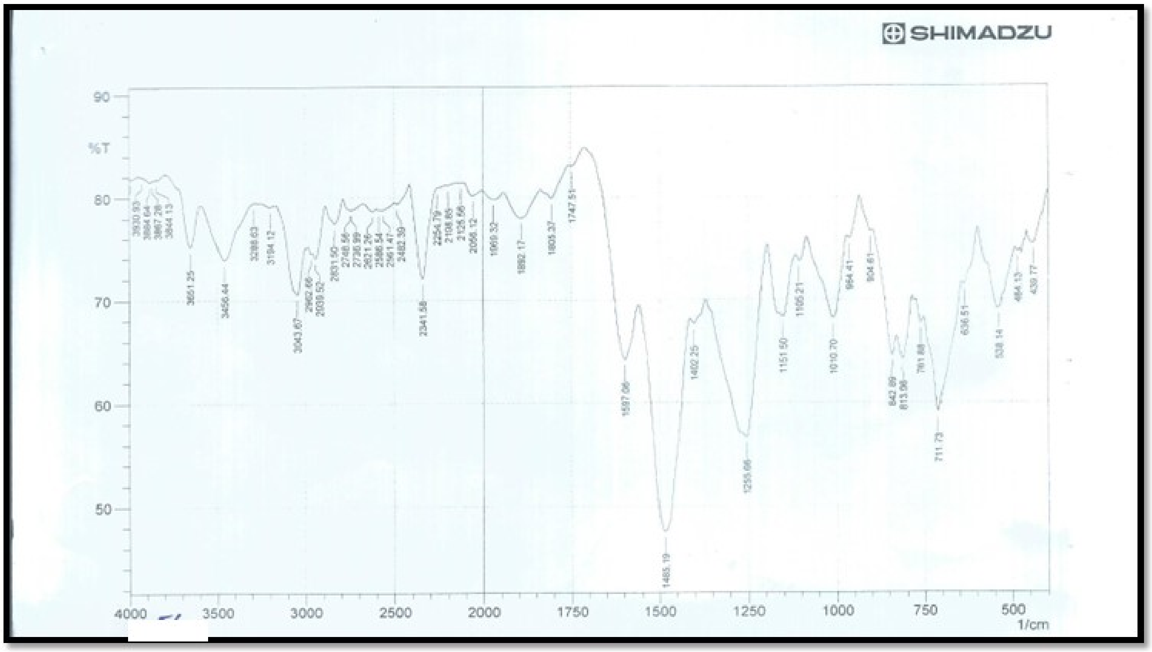

Fig. S8: FTIR Spectrum of *Zinc (II) complex (C_6)*

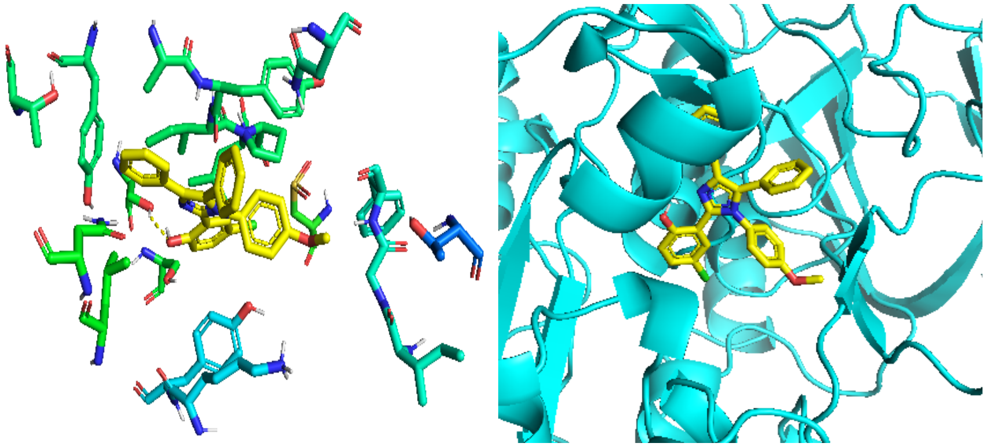

Ligand (**HL**)

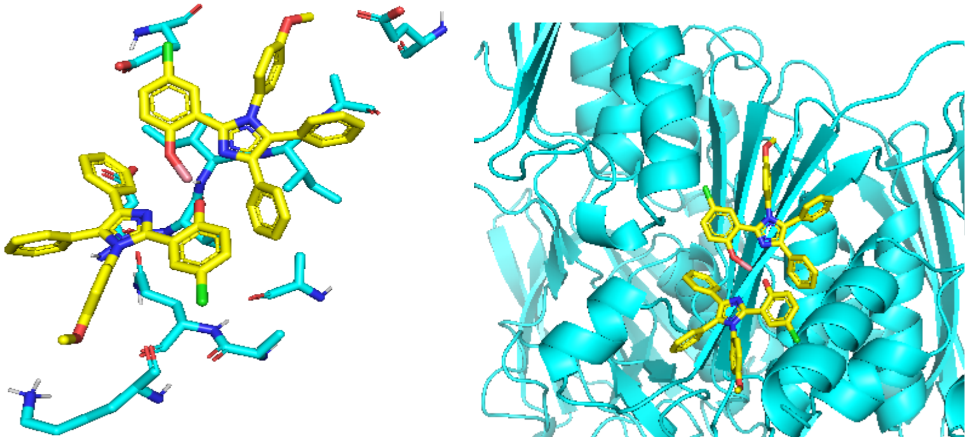

Co(II) complex ($\mathbf{C_1}$)

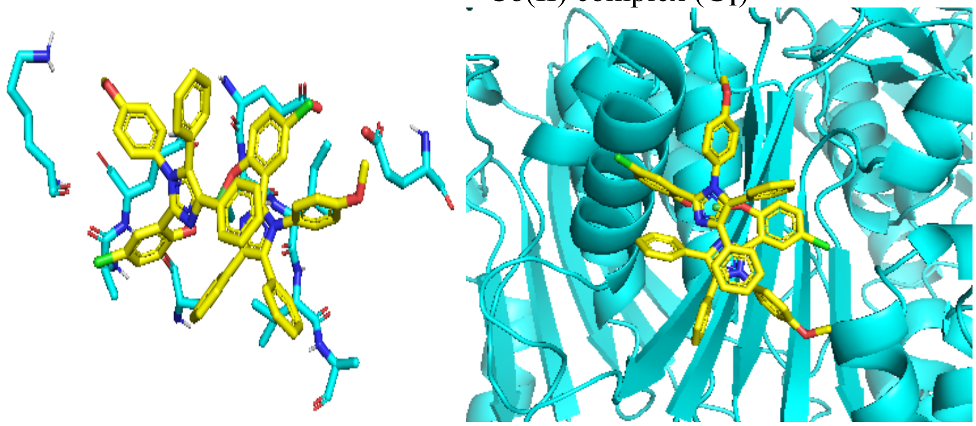

Ni(II) Complex ($\mathbf{C_2}$)

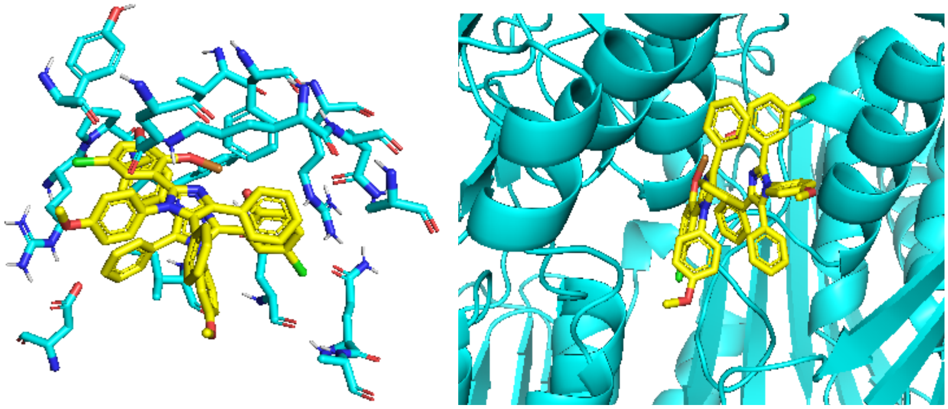

Cu(II) Complex ($\mathbf{C_3}$)

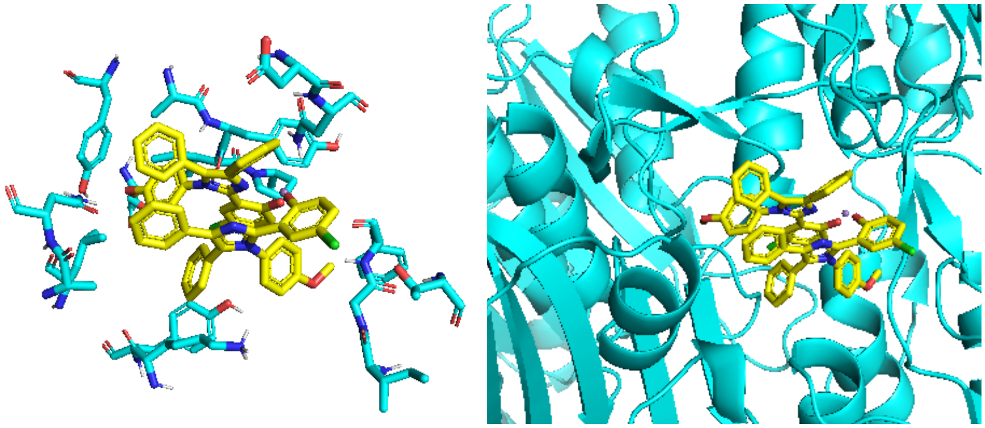

Mn(II) Complex ($\mathbf{C_4}$)

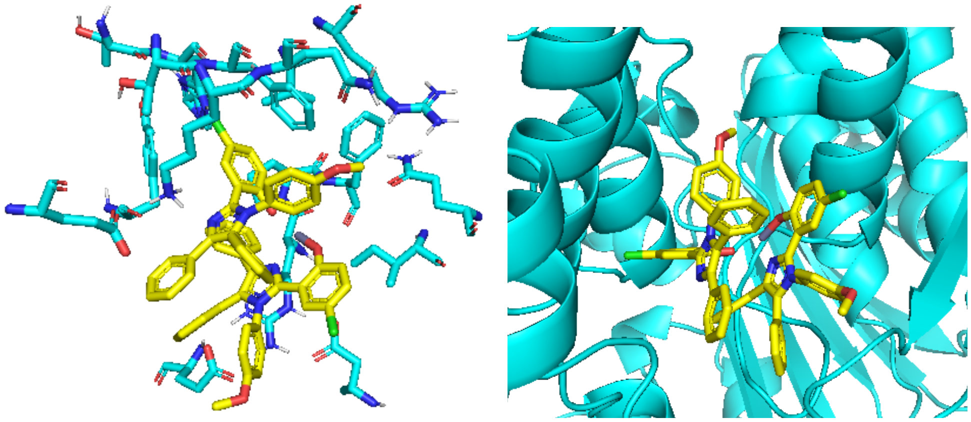

Zn(II) Complex ($\mathbf{C_5}$)

Fig. S9. Docking simulation of the interaction between synthesized compounds (yellow) and NADPH enzyme.

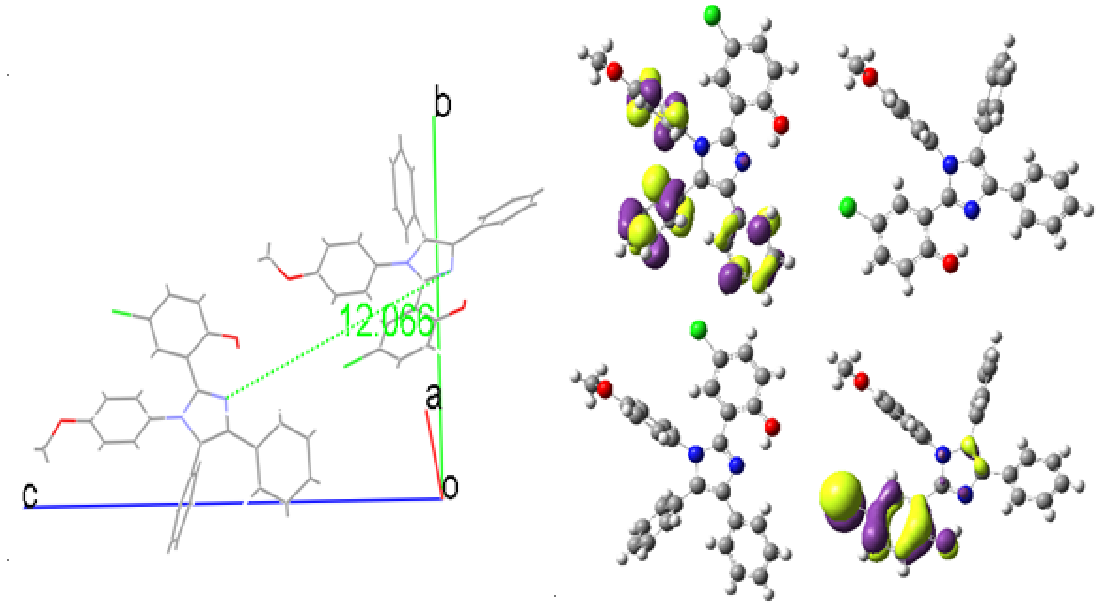

Fig. S10. Dimer packing (left) and its electron density distribution of FMOs (right, HOMO bottom while LUMO+1 top)

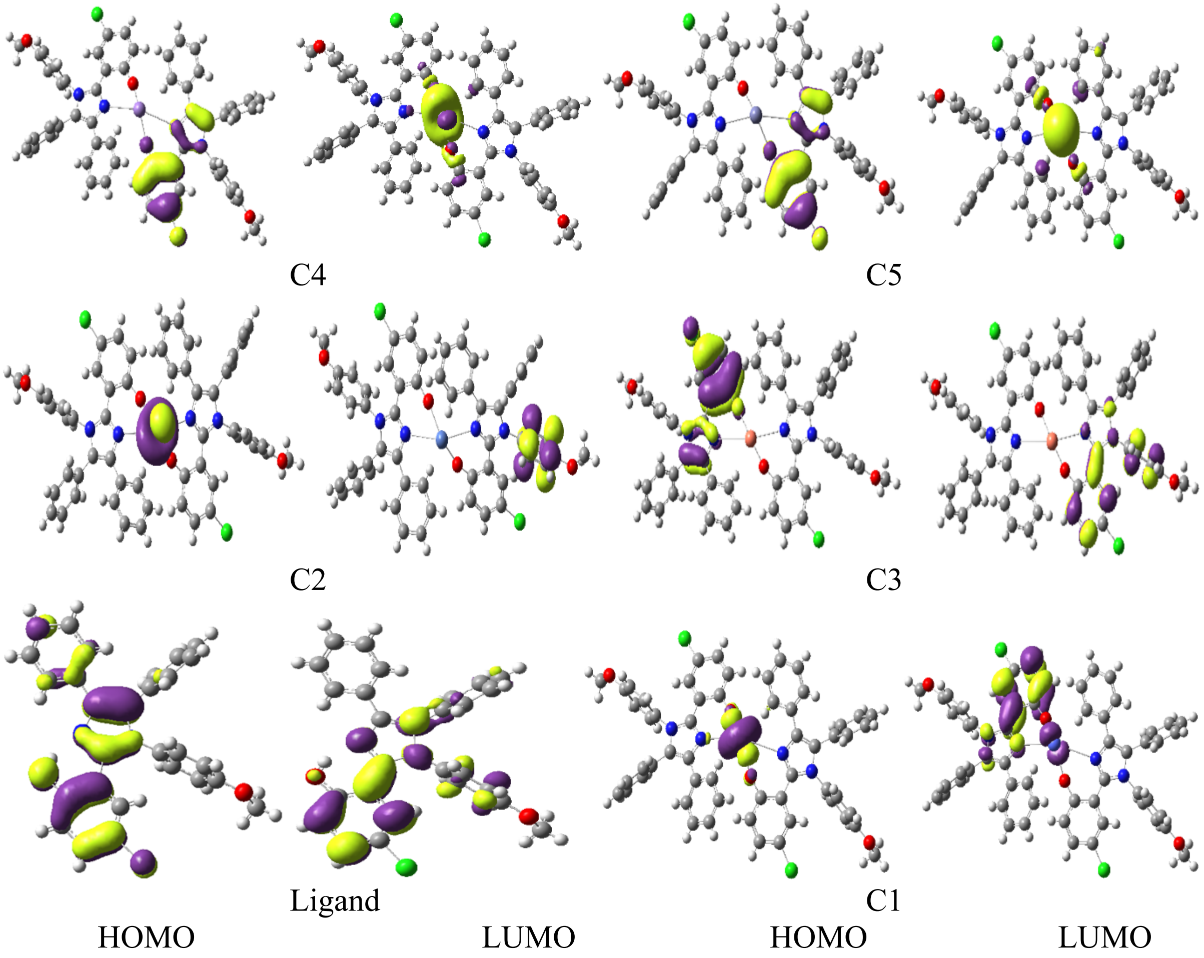

Fig. S11. The charge density distribution of HOMOs and LUMOs

Calculation of charge transport (CT) and transfer integral (*V*):

The proposed models of CT, the computational methodology is divided into additional parts, where electron or hole relics on a fragment and can hop from one part to another. By tight-binding calculation the hole or electron is approached in a single orbital, and imagined that just adjacent parts may couple. The calculations require precise data regarding electronic pairings about CT consequently hopping matrix or CT integrals or transfer integrals). The site energies (specific compound localized charge energy) considered with the function of the geometric conformation vis-à-vis nearby monomer. ADF allows to employ individual compounds molecular orbitals (MO) as a basis set in calculations maintained by one system containing two or higher molecules.

For the *V* calculations, we used single crystal structure where the adjacent intermolecular hopping paths were used. Usually, *V* was estimated by Koopmans's theorem and/or direct method. According to Koopmans's theorem *V* can be calculated by half HOMO/LUMO energies for hole and electron, respectively. This method was used to probe the transfer integrals of some conjugated systems. In another study it was noticed that if the dimer is not co-facially superimposed then site energy has to be considered. In another study, it was showed that direct method to calculate the *V* "site-energy rectifies frontier orbital (FO) splitting technique" is a handsome approach. Moreover, previously it was also showed that INDO overestimated the *V* that ill-used the "dimer energy splitting" while direct approach is rational one. *V* values of electron and hole were calculated by direct approach using the equations:

$$V\text{hole/electron} = \langle \phi_{LUMO/HOMO}^{0,site1} \,|\, \mathrm{F}^0 \,|\, \phi_{LUMO/HOMO}^{0,site2} \rangle = \langle \phi_{LUMO/HOMO}^{0,site1} \,|\, \mathrm{h}_{\mathrm{core}} \,|\, \phi_{LUMO/HOMO}^{0,site2} \rangle + \sum_{l(occ)} (\langle \phi_{LUMO/HOMO}^{0,site1} \phi_l^0 \,|\, \phi_{LUMO/HOMO}^{0,site2} \phi_l^0 \rangle - \langle \phi_{LUMO/HOMO}^{0,site1} \phi_{LUMO/HOMO}^{0,site2} \,|\, \phi_l^0 \phi_l^0 \rangle) \quad (2)$$

The equation 2 exhibited, *V* hole/electron is hole and electron transfer integrals, whereas $\phi_{LUMO/HOMO}^{0,site1}$ and $\phi_{LUMO/HOMO}^{0,site2}$ terms are the HOMOs/LUMOs for the two adjoining monomers. Fock operator is F^0. Hitherto it was clarified that "during excitons formation, the spin reliance of the charge recombination rates" may be supported through interchange. The SCF (self-consistent field) can be used to calculate MO discretely, partaking none interaction for both individual monomers. The equation 2, dimers Fock matrix along with two-electron integrals could be built for non-interacting MO. Fock matrix dimer was estimated via density matrix interacting MO and

non-interacting MOs. The non-interacting MO used to evaluate the Fock matrix and density matrix according to eq. 3:

$$F = SC\varepsilon C^{-1} \quad (3)$$

where, dimer overlap matrix “S”; the eigenvalue (C and ε) along with Kohn-Sham orbitals were developed from the zeroth-order Fock matrix with “Fock matrix diagonalizing withoutany self-consistent field iteration”. Previous work revealed that PW91 gave good results for the *V* values. The PW91/TZP level was adopted to evaluate the Fock matrix dimer from MO of unruffled monomer. The reorganization energy (λ) can be divided as $\lambda_{rel}^{(1)}$ and $\lambda_{rel}^{(2)}$ where first and second terms are geometry relaxation energies from charged to neutral state and neutral to charged state, respectively, see eq. 4:

$$\lambda = \lambda_{rel}^{(1)} + \lambda_{rel}^{(2)} \quad (4)$$

The λ can be evaluated as under [40].

$$\lambda = \lambda_{rel}^{(1)} + \lambda_{rel}^{(2)} = [E^{(1)}(V^{+/-}) - E^{(0)}(V^{+/-})] + [E^{(1)}(V) - E^{(0)}(V)] \quad (5)$$

where, the $E^{(0)}(V^{+/-})$ and $E^{(0)}(V)$ are the charged and neutral state energies, respectively. $E^{(1)}(V)$ and $E^{(1)}(V^{+/-})$ are the energies with a neutral (charged) state having optimized charged (neutral) geometry, respectively.
